# Temperature Variations in a Flexible Thermoelectric Module with an Evaporative Heat Sink

**DOI:** 10.3390/ma19010163

**Published:** 2026-01-02

**Authors:** Monika Jangas, Anna Dąbrowska, Łukasz Starzak, Bartosz Pękosławski, Agata Kmiecik, Marcin Jachowicz, Szymon Ordysiński

**Affiliations:** 1 Department of Personal Protective Equipment, Central Institute for Labour Protection-National Research Institute, Wierzbowa 48, 90-133 Lodz, Poland; andab@ciop.lodz.pl (A.D.); agkmi@ciop.lodz.pl (A.K.); majac@ciop.lodz.pl (M.J.); 2 Department of Microelectronics and Computer Science, Lodz University of Technology, Wólczańska 221 Building B18, 93-005 Lodz, Poland; lukasz.starzak@p.lodz.pl (Ł.S.); bartosz.pekoslawski@p.lodz.pl (B.P.); 3 Department of Safety and Health Management, Central Institute for Labour Protection-National Research Institute, Czerniakowska 16, 00-701 Warsaw, Poland

**Keywords:** cooling garment, thermoelectric cooling, Peltier effect, flexible thermoelectric module, thermoelectric module, heat sink

## Abstract

Exposure to hot microclimate constitutes a serious threat to human health, especially in environments where collective protection measures cannot be implemented. Despite technological advances, personal cooling solutions remain insufficient for long-term use. Thermoelectric modules (TEMs) offer a promising pathway for developing cooling garments. This paper deals with temperature variations in a cooling set composed of a TEM and an evaporative heat sink, for different supply currents. A special methodology was adopted that included the use of a skin model placed in a climatic chamber, and temperature sensors that allowed temperatures at several points to be recorded. After 30 min of operation, the cold side temperature of the TEM was approximately 3 °C to 4.5 °C lower than when the heat sink was absent and the TEM was not supplied. This is close to what thermal comfort requires and may become too small for longer operation or less favourable climatic conditions. Enhanced heat dissipation from the hot side is therefore essential for enabling TEMs to function effectively in wearable colling systems, which makes research on heat sinks other than evaporative ones necessary.

## 1. Introduction

### 1.1. Hot Microclimate

According to the European Survey of Enterprises on New and Emerging Risks (ESENER) [1] conducted by the European Union information agency for occupational safety and health (EU-OSHA), an average of 37% of establishments across the present European Union (EU) report physical risk factors related to heat, cold, or draught. This makes thermal risk one of the five most common physical hazards in EU workplaces (less frequent than prolonged sitting, repetitive hand or arm movements, lifting people or heavy loads, and accidents, but more frequent than exposure to chemical or biological substances, tiring or painful working positions, and loud noise). The relative position of the thermal risk has remained fairly stable across ESENER surveys (2014-2024) [2], although it has varied notably across countries. For instance, nearly 50% of enterprises in Germany have reported this risk, with similarly prominent levels in Finland and Latvia (46%), and in France and Norway (44%), while in some other countries the figure has been just over 20%.

Since the ESENER data are based on subjective assessment and do not account for the number of exposed workers, or exposure duration and severity, it is important to complement them with objective and more precise measurements. Few such sources are available, including a national survey conducted by the Polish Statistical Office which indicates that nearly 8% of employees exposed to occupational hazards are affected by hot or cold microclimates (more often to hot: 5%) [3]. The vast majority (over 91%) of people working in high temperatures are employed in industry, particularly in mining, quarrying, and manufacturing.

As scientists and experts predict more frequent and severe heatwaves and rising global temperatures due to climate change, occupational exposure to high temperatures is also expected to increase. This trend will possibly lead to increased mortality, reduced productivity and damage to infrastructure [4], posing a serious threat to the safety and health of workers in most parts of the world. The International Labour Organization (ILO) estimates that more than 70% of the global workforce (at least 2.4 billion workers) are currently exposed to excessive heat during some part of their work [5]. Moreover, this proportion has already increased from 65.5% in 2000 to 70.9% and is projected to rise further. Heat-related occupational risks contribute to nearly 19,000 deaths annually and result in the loss of over 2 million disability-adjusted life years (DALYs), due to the 22.87 million heat-attributable occupational injuries. Furthermore, as of 2020, 26.2 million people worldwide are living with chronic kidney disease associated with workplace heat stress.

The economic sectors at high risk include agriculture, construction, transport, emergency repair services, and tourism, particularly where work is physically demanding or carried out in poorly ventilated environments. High temperatures can cause greater heat stress in the workplace, which in turn can lead to an increase in heat-related illnesses (e.g., heat stroke, heat exhaustion), reduced tolerance to chemicals, and fatigue [6]. Exposure to hot microclimates affects both physiological and psychomotor functions of the human body [7]. It has been observed that as ambient temperature rises, it is more difficult to concentrate, the number of mistakes made increases, and the ability to perform physical work decreases [8,9,10]. In Spain, occupational injuries related to extreme heat account for 2.72% of all injuries [11,12].

### 1.2. Thermoelectric Modules

In many workplaces, collective protective measures against heat cannot be implemented due to the nature of the work performed or the technological process involved. Individual cooling is the only possible solution there. A promising direction for development in this area is the application of thermoelectric modules.

Thermoelectric effects relate to direct conversion between electrical and thermal energy. The generation of a temperature difference by an electric current is called the Peltier effect. The elementary components intended for the creation and practical use of thermoelectric effects are called thermoelectric cells [13]. The core of such a cell is usually two legs made of semiconductors of distinct types, N (electron-majority one) and P (hole-majority one). Current is carried by electrons or holes whose energy is different in dissimilar materials. As these carriers pass from one material to another, this energy difference is either released as heat (heating) or absorbed as heat (cooling), depending on the direction of current [14,15].

A single cell can process only a small amount of heat, so multiple cells are connected both electrically (in series) and thermally (in parallel) to form thermoelectric modules (TEMs). In conventional TEMs, cells are electrically isolated from the environment by ceramic plates of low electrical conductivity and high thermal conductivity [13]. These plates make TEMs thick, heavy, rigid, and brittle, and prevent them from adhering to curved surfaces. This limits their use in wearable electronics and has motivated the development of flexible TEMs [16,17,18,19,20,21]. In contrast to conventional ones, they are light, can adjust to various shapes such as the human body, have lower heat losses and higher energy conversion efficiency. They can be embroidered, since they are made of semiconducting polymer yarns [22]. A more common solution, however, consists of embedding conventional thermoelectric cells in an elastic filler [23,24]. Flexible ferroelectric thick-film structures that exhibit electrocaloric capabilities have also been studied for cooling applications [25] but the relatively high required voltage limits their suitability for wearable solutions [26].

This paper relates to the research whose first results were presented in [27]. That article concerned the effect of supply current on the heat flux transported by flexible TEMs of different dimensions. The aim of this work is to analyze the time variation in temperatures in a TEM and heat sink set as well as the influence of supply current thereon. The temperatures analyzed are those on the cold side of the TEM, at the TEM–heat sink interface, and on the outer surface of the heat sink.

## 2. Materials and Methods

### 2.1. Tested Object

**Figure 1:**
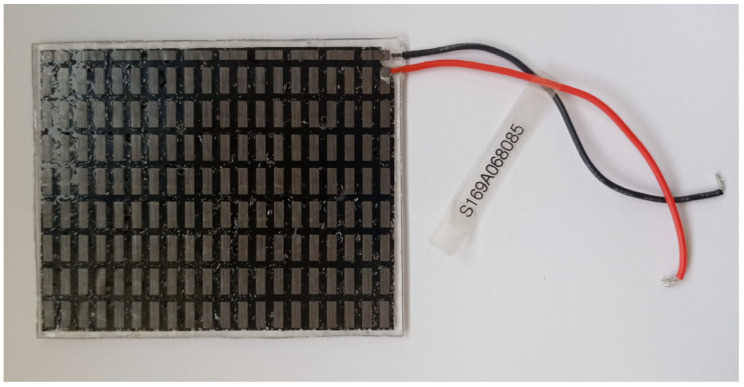
Top view of the S169A068085 flexible TEM [27].

An S169A068085 flexible TEM (Figure 1) from FTED Co., Ltd. (formerly TEGway Co., Ltd., Daejeon, Republic of Korea) was examined. It was selected based on the results presented in [27] as it minimized the number of modules per heat flow rate while providing the second-best maximum average heat flux density and the second-best corresponding coefficient of performance (COP) among all the TEMs tested. Its parameters are listed in Table 1 while its photograph is shown in Figure 1.

The TEM under test is composed of 169 cells based on bismuth and tellurium compounds (Bi-Te) arranged in an array. The N-type and P-type legs are adhered to metal film electrodes using an electrically conductive adhesive. Voids between electrodes and thermoelectric materials are filled with low thermal conductivity elastic polymer (polydimethylsiloxane, PDMS) foam to prevent oxidation and to ensure high mechanical strength and flexibility. The module is covered with two thermally conductive thin-film contact layers to minimize thermal resistance [28,29].

The TEM was tested with a flame-retardant multi-shield fabric applied on its cold side, consistently with its planned application in protective clothing. The material used was composed of Nomex (93%), Kevlar (5%), and antistatic fibre (2%). Its parameters are listed in Table 2.

**Figure 2:**
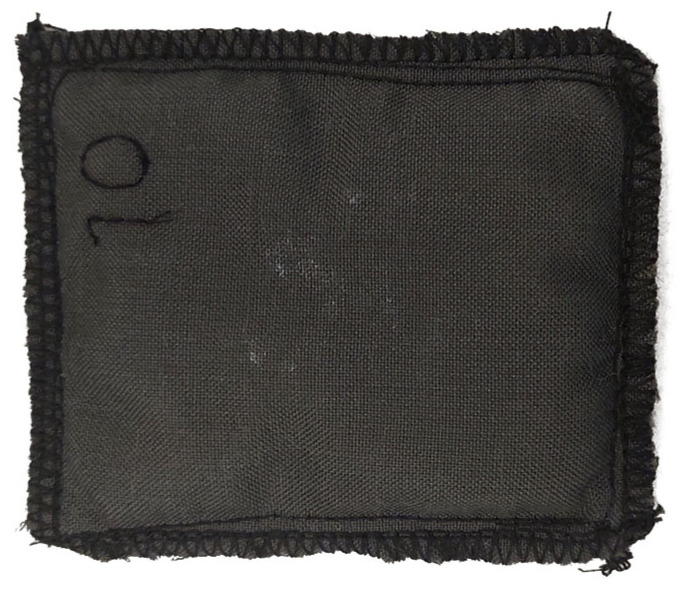
Top view of the heat sink.

On the hot side of the TEM, a heat sink was applied. It was made of Technical Absorbents SAF^TM^ Fabric 2644 superabsorbent non-woven fabric (surface mass: 345 g/m^2^) (Technical Absorbents Limited, Grimsby, United Kingdom) sewn on both sides with the multi-shield fabric indicated above. Its photograph is shown in Figure 2. The heat sink dissipates heat by evaporation after being soaked with water. Its dimensions corresponded to those of the TEM, i.e., 85 mm × 68 mm.

### 2.2. Testing Methodology

#### 2.2.1. Research Apparatus

**Figure 3:**
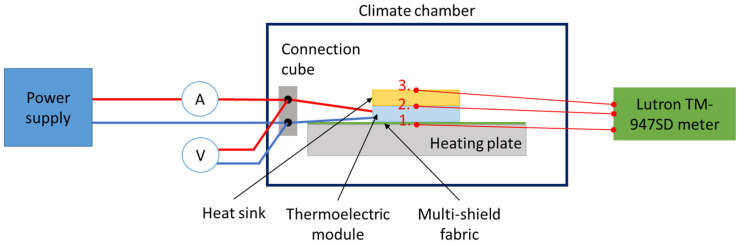
Test setup diagram with temperature measurement points marked as follows: 1–on the cold side of the TEM; 2–at the TEM–heat sink interface; 3–on the outer surface of the heat sink.

The testing methodology developed earlier [27,30] was applied. It involved the use of a skin model in the form of an electrically heated plate whose heating power can be recorded. The skin model together with the tested materials were placed in a WK11 340 climatic chamber (Weiss Technik GmbH, Reiskirchen, Germany) which ensured controlled ambient conditions during the tests. Temperatures in the system were measured using three TP-01 type K probes (Lutron Electronic Enterprise Co., Ltd., Taipei, China) with a measurement range of −40 °C to 250 °C, and a TM-947SD temperature meter (Lutron Electronic Enterprise Co., Ltd., Taipei, China) allowing data recording on a memory card. Temperature measurement accuracy was ±(0.4% of the reading + 0.5 °C). A diagram of the test setup is shown in Figure 3.

TEM current and voltage were measured using digital multimeter CD772 (Sanwa Electric Instrument Co., Ltd., Tokyo, Japan).

#### 2.2.2. Research Conditions

The tests were conducted at a constant and equal air and heated plate temperature of 35 °C, a constant relative humidity of 40%, and an air movement velocity of 1 m/s. The isothermal conditions between the ambient and the skin model were forced to eliminate any heat exchange except through the TEM so that the recorded heating power of the plate was identical with the heat flow rate on the cold side of the TEM [27].

#### 2.2.3. Measured Parameters

Temperatures in three locations were measured as follows:

- with probe 1, on the cold side of the TEM, allowing the cooling efficiency of the TEM and heat sink set to be evaluated;
- with probe 2, at the TEM–heat sink interface, allowing the efficiency of heat removal by the heat sink to be evaluated; and
- with probe 3, on the outer surface of the heat sink.

**Figure 4:**
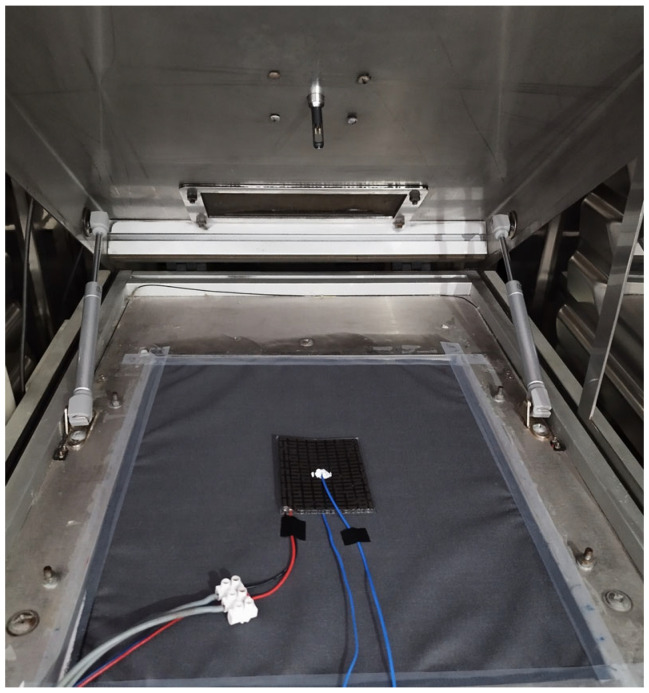
The TEM under test conditions, when placed on the multi-shield fabric covering the skin model, with supply and voltage measurement wires (red and grey), and temperature probe wires (blue) visible [27].

To decrease thermal resistance between the probes and the relevant object or objects, “H” thermally conductive silicone paste was applied (Figure 4).

TEM current and voltage were measured and used to calculate electric power. Cold side heat flow rate was measured as skin model heating power. Electric power and cold side heat flow rate were averaged over the entire test duration and the resulting values were used to evaluate the coefficient of performance of the TEM and heat sink set in the way detailed in [27].

#### 2.2.4. Research Procedure

Tests were conducted for the TEM supply current varying from 0.05 A to 0.55 A with a step of 0.05 A. To minimize the influence of heat sink wear, a new item was prepared and used in each test. The heated plate of the skin model was fully covered with the multi-shield fabric, and the TEM was placed on the latter, with its cold side facing the plate (Figure 4). The climatic chamber was then closed and the conditions specified in Section 2.2.2 were set. Prior to testing, each heat sink was soaked for 1 min with distilled water; the water’s constantly monitored temperature was approximately 20 °C. The TEM power supply was set to the appropriate current but initially held inactive.

The test was started once the setup reached a steady state, as indicated by the skin model heating power having stabilized at zero, but no earlier than 30 min after closing the chamber. At this point, the chamber door was opened, the soaked heat sink was placed on the TEM, the door was closed, and the TEM power supply was activated in its constant current mode. These operations were conducted as quickly as possible to minimize their impact on results. The duration of each test was 30 min, which corresponded to the stabilization time of the skin model heating power.

## 3. Results

**Figure 5:**
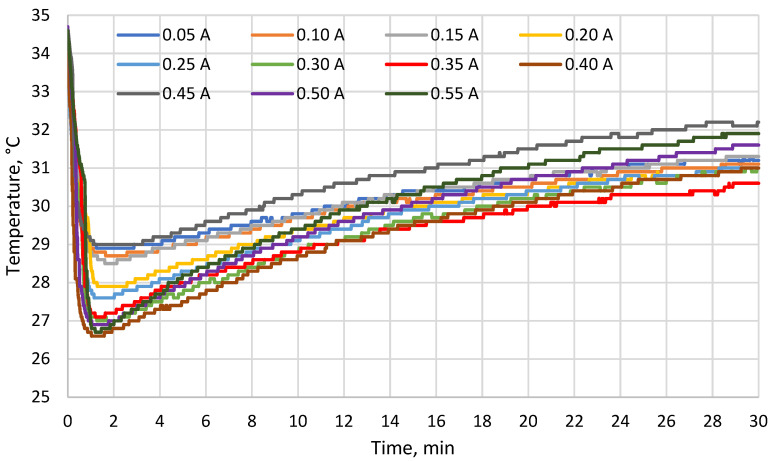
Temperature on the cold side of the TEM (probe 1) as a function of time for different supply currents.

**Figure 6:**
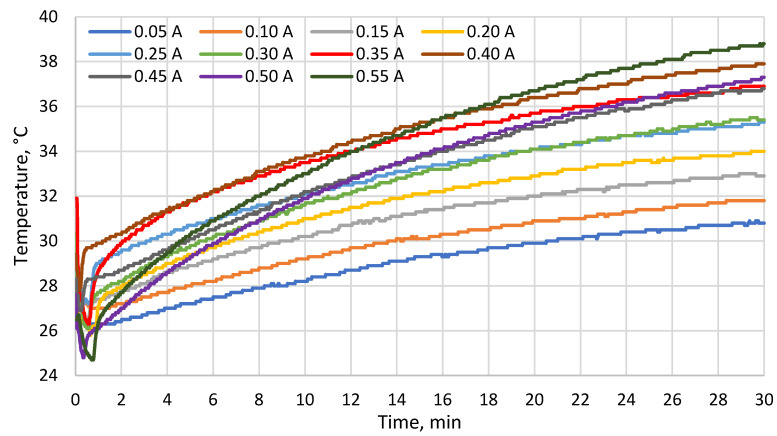
Temperature at the TEM–heat sink interface (probe 2) as a function of time for different supply currents.

**Figure 7:**
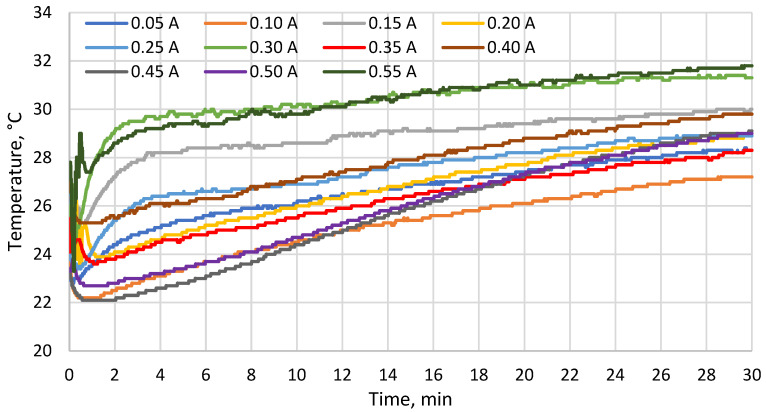
Temperature on the outer surface of the heat sink (probe 3) as a function of time for different supply currents.

Figure 5, Figure 6 and Figure 7 show the temperatures measured with probes 1–3, respectively. Starting from approx. 1 min, all these temperatures were increasing. The results show that at the end of the test, there were correlations between the TEM supply current and the cold side temperature of the TEM (which decreased with current up to 0.35 A, see Figure 5) as well as the temperature at the TEM–heat sink interface (which increased with current, see Figure 6). The temperature difference between the cold side (Figure 6) and the hot side (Figure 7) of the TEM reached around 7 °C at maximum.

A detailed heat flow analysis of the system under test conditions was presented in [27]. The COP calculated for the TEM—tested in this paper from measurement data averaged over the entire test duration—was 4.0 (0.3) at the current of 0.26 A which corresponded to an estimated maximum cold side heat flux.

## 4. Discussion

Within the first two minutes of the test, the cold side temperature of the TEM (Figure 5) dropped by 5 °C to 7 °C (depending on the supply current) with respect to its initial value equal to the skin model temperature of 35 °C. For comparison, Gao et al. [31] developed a thermoelectric cooler that lowered the temperature by 9.1 °C indoors and by 6.5 °C outdoors. At the end of the test, the cold side temperature was still lower than that of the skin model, but the difference between them decreased to ca. 3 °C to 4.5 °C. These results indicate that the evaporative heat sink used lost its ability to remove heat from the hot side of the TEM due to the limited heat capacity of water and the resulting limited heat accumulation.

**Figure 8:**
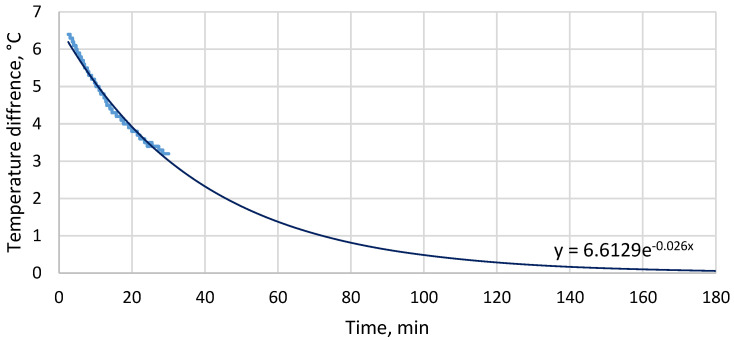
Calculated (thick line) and estimated (thin line) temperature difference between the skin model and the cold side of the TEM as a function of time for the supply current of 0.25 A (the calculated values are based on the rising portion of the measured cold side temperature; the estimated values have been obtained by least squares fitting of the exponential function whose equation is shown in the graph).

According to a previous study of a cooling garment for professional use [32], a reduction in skin temperature of 1.0 °C to 2.7 °C is sufficient for maintaining the thermal comfort sensation of a user performing an intense physical activity at the ambient temperature of 25 °C. The reduction of 3 °C observed in this test was near the upper bound of this range and Figure 5 shows that the relevant temperature was still increasing at the end of each test. If the temperature difference between the skin model and the cold side of the TEM is calculated and extrapolated with an exponential function (Figure 8), the estimated value is less than 0.1 °C after 180 min. Moreover, true skin temperature would be higher than the cold side temperature of the TEM due to the thermal resistance between them. These results show that if the operating time is much longer than 30 min, or temperature and/or humidity are higher than those applied in these tests, the cooling performance of the investigated TEM and heat sink set is likely to fall below what is required for thermal comfort. The heat sink applied is therefore insufficient to ensure the operation of a cooling garment for an entire work shift duration without re-soaking the heat sink or replacing it with a freshly soaked item.

Among the supply currents applied in this study, 0.25 A is the closest one to 0.26 A which was estimated in [27] to yield maximum heat flux for the TEM analyzed in this paper. At 0.25 A, the minimum cold side temperature of the TEM was ca. 27.5 °C (7.5 °C less than the skin model temperature), while at the end of the test, the cold side temperature was ca. 31 °C (4 °C less than the skin model temperature). At the same current, the minimum temperature at the TEM–heat sink interface was ca. 27.5 °C while the maximum one was ca. 35 °C. The minimum temperature on the outer surface of the heat sink was ca. 23.5 °C while the maximum one was ca. 29 °C. As compared to other current values applied, these results were in the mid, mid-low or mid-high part of the range, depending on the location and time. The maximum cold side heat flux estimated in [27] therefore does not coincide with the lowest temperatures recorded.

The value of COP indicated in Section 3 was the second highest among the TEMs investigated in [27], at the estimated current of maximum heat flux. As mentioned in [27], this value does not characterize the TEM alone but the entire set composed of the TEM, the heat sink, and the multi-shield fabric.

## 5. Conclusions

The cold side temperature of the TEM at the end of each test decreased with increasing supply current up to 0.35 A. The temperature at the module–heat sink interface at the end of each test rose with current. During each test, both these temperatures were increasing for most of the time due to the heating of the water contained in the heat sink. The temperature difference between the cold and hot sides of the TEM reached ca. 7 °C at maximum.

In the initial phase of the test, the cold side temperature of the TEM was lower by approximately 5 °C to 7 °C (depending on the supply current) than the skin model temperature. At the end of the test, however, this difference was of only 3 °C to 4.5 °C, indicating that the performance of the heat sink used decreased in time to a point where it is insufficient for longer operation or less favourable climatic conditions.

Therefore, further work will involve computer modelling and simulation of heat removal from the hot side using heat sinks that employ heat removal mechanisms different than evaporation. This will enable a suitable heat sink to be selected for the planned application of TEMs in cooling garments. It should be noted that flexibility is a key feature of a heat sink in this case. Flexible heat sinks, including ones integrated with TEMs, have been reported recently [31,33,34].

## Figures and Tables

**Table 1:** Parameters of the TEGway flexible thermoelectric module S169A068085 [27].

Parameter and Unit	Value
Length, mm	85
Width, mm	68
Thickness, mm	6
Rated current, A	6
Rated voltage, V	22
Rated heat flow rate, W	76.5

**Table 2:** Multi-shield fabric parameters.

Parameter and Unit	Value and Tolerance
Thickness, mm	0.360 ± 0.007
Surface mass, g/m^2^	154.2 ± 1.4
Thermal resistance, m^2^ × K/W	0.016 ± 0.001
Water vapour resistance, m^2^ × Pa/W	3.281 ± 0.171

## Data Availability

The original contributions presented in this study are included in the article. Further inquiries can be directed to the corresponding author.
